# Phenotypic and Genotypic Drug Resistance of *Mycobacterium tuberculosis* Strains Isolated from HIV-Infected Patients from a Third-Level Public Hospital in Mexico

**DOI:** 10.3390/pathogens13020098

**Published:** 2024-01-23

**Authors:** Daniel Valencia-Trujillo, Amanda Marineth Avila-Trejo, Rocío Liliana García-Reyes, Luis Narváez-Díaz, Mario Alberto Mújica-Sánchez, Addy Cecilia Helguera-Repetto, Eduardo Becerril-Vargas, Mónica Maribel Mata-Miranda, Sandra Rivera-Gutiérrez, Jorge Francisco Cerna-Cortés

**Affiliations:** 1 Departamento de Microbiología, Escuela Nacional de Ciencias Biológicas, Instituto Politécnico Nacional, Ciudad de México 11340, Mexico; vatd921205@hotmail.com (D.V.-T.); rlgarciar@ipn.mx (R.L.G.-R.); srivera@ipn.mx (S.R.-G.); 2 Servicio de Microbiología Clínica, Instituto Nacional de Enfermedades Respiratorias, Ciudad de México 14080, Mexico; nluisnd0304@hotmail.com (L.N.-D.); mario.mugica1@gmail.com (M.A.M.-S.); edobec.var@gmail.com (E.B.-V.); 3 Escuela Militar de Medicina, Centro Militar de Ciencias de la Salud, Secretaría de la Defensa Nacional, Ciudad de México 11200, Mexico; mmcmaribel@gmail.com; 4 Laboratorio de Bioquímica Farmacológica, Departamento de Bioquímica, Escuela Nacional de Ciencias Biológicas, Instituto Politécnico Nacional, Ciudad de México 11340, Mexico; ammarat.gdds@gmail.com; 5 Departamento de Inmunobioquímica, Instituto Nacional de Perinatología Isidro Espinosa de los Reyes, Ciudad de México 11000, Mexico; ceciliahelguera@yahoo.com.mx

**Keywords:** *Mycobacterium tuberculosis*, *tuberculosis*, drug susceptibility test, HIV-infected patients

## Abstract

Background: Drug-resistant tuberculosis (TB) is associated with higher mortality rates in patients with human immunodeficiency virus (HIV). In Mexico, the number of deaths due to TB among the HIV-positive population has tripled in recent years. Methods: Ninety-three *Mycobacterium tuberculosis* strains isolated from the same number of HIV-infected patients treated in a public hospital in Mexico City were studied to determine the drug resistance to first- and second-line anti-TB drugs and to identify the mutations associated with the resistance. Results: Of the 93 patients, 82.7% were new TB cases, 86% were male, and 73% had extrapulmonary TB. Most patients (94%) with a CD4 T-lymphocyte count <350 cells/mm^3^ were associated with extrapulmonary TB (*p* <0.0001), whilst most patients (78%) with a CD4 T-lymphocyte count >350 cells/mm^3^ were associated with pulmonary TB (*p* = 0.0011). Eighty-two strains were pan-susceptible, four mono-resistant, four poly-resistant, two multidrug-resistant, and one was extensively drug-resistant. In the rifampicin-resistant strains, *rpoB* S531L was the mutation most frequently identified, whereas the *inhA* C15T and *katG* S315T1 mutations were present in isoniazid-resistant strains. The extensively drug-resistant strain also contained the mutation *gyrA* D94A. Conclusions: These data highlight the need to promptly diagnose the drug resistance of *M. tuberculosis* among all HIV-infected patients by systematically offering access to first- and second-line drug susceptibility testing and to tailor the treatment regimen based on the resistance patterns to reduce the number of deaths in HIV-infected patients.

## 1. Introduction

Tuberculosis (TB) is caused by the bacillus *Mycobacterium tuberculosis* (*M. tuberculosis*) and is a communicable disease that is a major cause of ill health and one of the leading causes of death worldwide [1]. The infection is almost exclusively transmitted airborne when an individual inhales live bacteria released in aerosolized microdroplets/droplet bioaerosols that are generated when an individual with active and advanced symptomatic pulmonary TB disease laughs, sneezes, coughs, or talks. Such aerosols can remain airborne and infectious for several hours, be carried in the air, and accumulate in poorly ventilated environments [2]. The lung is the most commonly affected organ in TB infection in the immunocompetent host, with estimates of lung involvement in subjects with active TB of 79% to 87% [3]. The estimates of lung involvement are similar in immunocompromised hosts, such as those with human immunodeficiency virus (HIV) infection; however, these individuals are also more likely to also have extrapulmonary disease [3].

Tuberculosis is a preventable and usually curable disease. However, the WHO reported that an estimated of 10.6 million people developed TB worldwide in 2022 [1]. Further, TB is the world’s second leading cause of death from a single infectious agent, after coronavirus disease (COVID-19), causing an estimated 1.3 million deaths [1]. Mexico has a population of more than 128 million inhabitants and, according to the global TB report 2023, 36,000 new cases of TB were reported in Mexico in 2022, with an incidence of 28/100,000 inhabitants and 4900 deaths [4].

Drug-resistant TB continues to be a public health threat. Resistance to rifampicin, the most effective first-line drug, is of greatest concern [5]. Tuberculosis that is resistant to rifampicin and isoniazid is defined as multidrug-resistant TB (MDR-TB). Both MDR-TB and rifampicin-resistant TB (RR-TB) require treatment with second-line drugs [1]. In 2022, the estimated proportion of people with TB who had MDR/RR-TB was 3.3% among new cases and 17% among those previously treated [1]. Recently, the WHO reported that an estimated 410,000 people developed MDR/RR-TB in 2022. In Mexico, the MDR/RR-TB incidence increased from 760 in 2019 to 1300 cases in 2022, with an actual incidence of 1.0 per 100,000 inhabitants. The proportion of new cases with MDR/RR TB was 2.6%, which increased to 15% in previously treated cases [4].

People living with HIV are 16 times more likely to fall ill with TB than those without HIV, and TB is the leading cause of death among people with HIV. In these patients, HIV and TB form a lethal combination, each speeding the other’s progress. Without proper treatment, 45% of HIV-negative people with TB on average and nearly all HIV-positive people with TB will die. In 2022, approximately 167,000 people died of HIV-associated TB [6]. In Mexico, there are currently 370,000 people living with HIV [7], and the number of deaths due to TB among the HIV-positive population has tripled in recent years, going from 760 deaths in 2019 to 2000 in 2022 [4]. Drug-resistant TB is often associated with higher mortality rates in patients living with HIV [8,9]. Therefore, determining the drug resistance profiles of the *M. tuberculosis* strains that are affecting these patients is crucial to provide adequate therapeutic management to reduce the number of deaths in this population.

In Mexico, there is limited information about the prevalence of drug-resistant TB in HIV-infected patients. In this context, the aims of this study were to evaluate the drug-resistance patterns to first- and second-line anti-TB drugs and to determine the mutations related to drug-resistance in *M. tuberculosis* strains from a group of HIV-positive patients treated in a third-level public hospital in Mexico City, with the overall aim to advocate for and implement effective control strategies.

## 2. Materials and Methods

### 2.1. Sample Collection and Study Population

**Figure 1:**
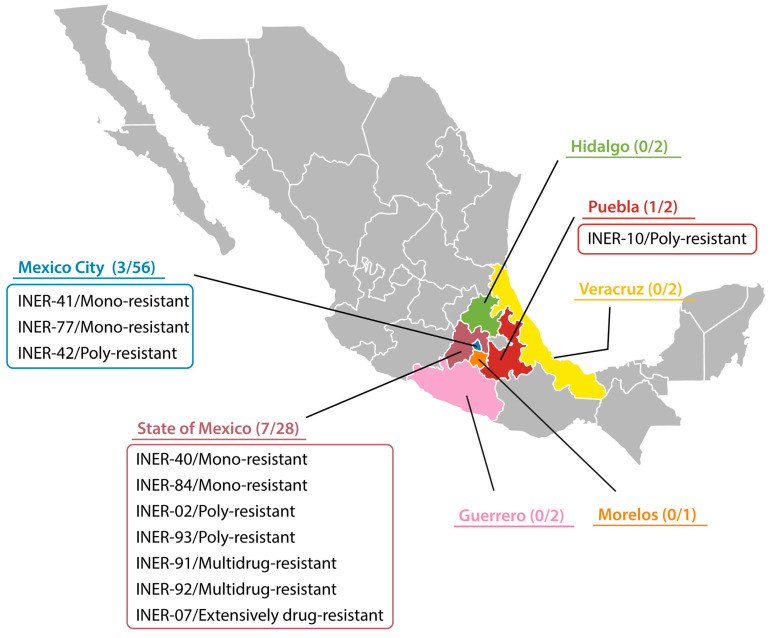
Geographical localization of each drug-resistance pattern within Mexico. The quotient represents the number of drug-resistant *M. tuberculosis* strains divided by the total number of strains collected.

*Mycobacterium tuberculosis* strains were isolated from HIV-infected patients from seven different states of Mexico (Figure 1) without any other comorbidities and treated in the National Institute of Respiratory Diseases “Ismael Cosio Villegas” (Mexico City) between January 2014 and December 2019. Mycobacteria were isolated from both pulmonary and extrapulmonary samples. Demographic and clinical data were recovered from the clinical files of each patient and included the state of residence, age, sex, occupation, level of education, clinical sample, type of TB (pulmonary or extrapulmonary), and CD4 T-lymphocyte count.

### 2.2. DNA Isolation and M. tuberculosis Identification

Biological samples were taken as part of routine diagnoses. Samples were decontaminated using Petroff’s modified method [10]. An aliquot was inoculated into Middlebrook 7H9 broth (Becton Dickinson) and incubated in the BD BACTEC™ MGIT™ automated mycobacterial detection system. Blood and bone marrow samples were not decontaminated and were directly inoculated into the BD BACTEC Myco/F Lytic culture vials, which were incubated in the BD BACTEC™ FX blood culture system. The DNA was extracted following the GenoType MTBC manufacturer’s instructions (HAIN Lifescience, Nehren Germany) and subjected to the GenoType MTBC test to ensure the reliable identification of *M. tuberculosis*.

### 2.3. First- and Second-Line Drug Susceptibility Testing and Search of Associated Mutations among Resistant M. tuberculosis Strains

Drug susceptibilities were tested in all *M. tuberculosis* strains according to the technical guide for the diagnosis of TB 2018 [11]. Susceptibility testing for the first-line drugs was performed using the antimicrobial susceptibility test carriers for streptomycin (STR) (1 and 4 μg/mL), isoniazid (INH) (0.1 and 0.4 μg/mL), rifampicin (RIF) (1 μg/mL), ethambutol (EMB) (5 and 8 μg/mL), and pyrazinamide (PZA) (100 μg/mL), using the fluorometric method (BACTEC^TM^ MGIT^TM^ 960^®^, Becton-Dickinson, Sparks, MD, USA). If the *M. tuberculosis* strains were resistant to rifampin and/or both concentrations of isoniazid, those strains were subjected to susceptibility tests for second-line drugs: amikacin (AMK) (1.0 and 2 μg/mL), capreomycin (CAP) (2.5 and 5.0 μg/mL), ethionamide (ETA) (5.0 μg/mL), kanamycin (KAN) (2.5 μg/mL), moxifloxacin (MOX) (1 and 2.0 μg/mL), and ofloxacin (OFX) (2 μg/mL). *Mycobacterium tuberculosis* strains were classified as pan-susceptible if they were susceptible to all the drugs tested. Drug resistance was defined as the resistance to one or more drugs. Mono-resistance was defined as resistance to only one drug and susceptibility to others. Poly-resistance was defined as resistance to multiple drugs, including either RIF or INH but not both RIF and INH. The MDR-TB refers to *M. tuberculosis* strains resistant to at least two key first-line anti-TB drugs: INH and RIF simultaneously. Extensively drug-resistant TB (XDR-TB) was defined as MDR-TB plus resistance to at least one of the fluoroquinolones (MOX and OFX) and one of the second-line injectable drugs (KAN, AMK, and CAP). *Mycobacterium tuberculosis* strain H37Rv, a strain susceptible to all drugs, was included as a control in all experiments. The *M. tuberculosis* strains resistant to any antibiotics were evaluated for search mutations using the kits GenoType MTBDRplus version 1.0 and GenoType MTBDRsl version 1.0, following the manufacturer’s instructions (Hain Lifescience, Nehren, Germany). The GenoType MTBDRplus assay allows the identification of mutations that confer resistance to INH (*rpoB* gene) and RIF (*katG* gene and *inhA* operon region) in *M. tuberculosis* strains, whereas GenoType MTBDRsl detects mutations in the *gyrA*, *rrs*, and *embB* genes and, therefore, resistance to fluoroquinolones, AMK/CAP, and EMB, respectively.

### 2.4. Spoligotyping of Drug-Resistant M. tuberculosis Strains

Spoligotyping was carried out following standard techniques [12,13]. The direct repeat (DR) region was amplified using oligonucleotides DRa (5′-GGTTTTGGGTCTGACGAC-3′, biotinylated) and DRb (5′-CCGAGAGGGGACGGAAAC’-3′). Labeled amplification products were used as a probe for hybridization with 43 synthetic spacer oligonucleotides covalently bound to a membrane (Isogen Biosciences B.M., Maarssen, The Netherlands). Each oligonucleotide corresponded to a known spacer sequence. PCR product bound after hybridization was detected by streptavidin-horseradish peroxidase-enhanced chemiluminescence and the membrane exposed to a chemiluminescence system, followed by exposure to X-ray film (Amersham, Little Chalfont, England) according to the manufacturer’s instructions. Spoligotypes were reported using an octal code [14]. Analysis of spoligotypes was performed using Bionumerics software version 5.5 (Applied Maths, Kortrijk, Belgium). *Mycobacterium tuberculosis* H37Rv was used as a control. Lineage and sublineage were assigned according to the SITVIT2 database (http://pasteur-guadeloupe.fr:8081/SITVIT2/ accessed on 10 July 2023) [15].

### 2.5. Statistical Analysis

The data from the patients included in the study were analyzed using descriptive and analytical statistics software (Centers for Disease Control and Prevention, Atlanta, GA, USA). To identify factors associated between TB with sociodemographic and clinical characteristics, Fisher’s exact test was used. Statistical analysis was carried out using Epi Info^TM^ version 7.2.5.0. Statistical significance was determined at *p* < 0.05.

## 3. Results

### 3.1. Sociodemographic and Clinical Characteristics of the Patients

In this study, we analyzed 93 *M. tuberculosis* strains isolated from the same number of HIV-infected patients from seven different states of Mexico (Figure 1). Of these 93 patients, 77 (82.7%) were new TB cases. Most (90%) patients came from Mexico City (60%) and the State of Mexico (30%). Patient age ranged between 7 and 65 years, with an average of 36 years. In this cohort, 80 (86%) male and 13 (14%) female individuals were included. A total of 43 (46%) patients were unemployed or disabled. Most patients (69%) had no or basic education. Sixty-eight (73%) patients had extrapulmonary TB, and the remaining had pulmonary TB. Regarding the CD4 T-lymphocyte count, most patients (94%) with <350 cells/mm^3^ were associated with extrapulmonary TB (*p* < 0.0001), whilst most patients (78%) with >350 cells/mm^3^ were associated with pulmonary TB (*p* = 0.0011) (Table 1).

### 3.2. Drug-Resistance Patterns to First- and Second-Line Anti-TB Drugs

**Figure 2:**
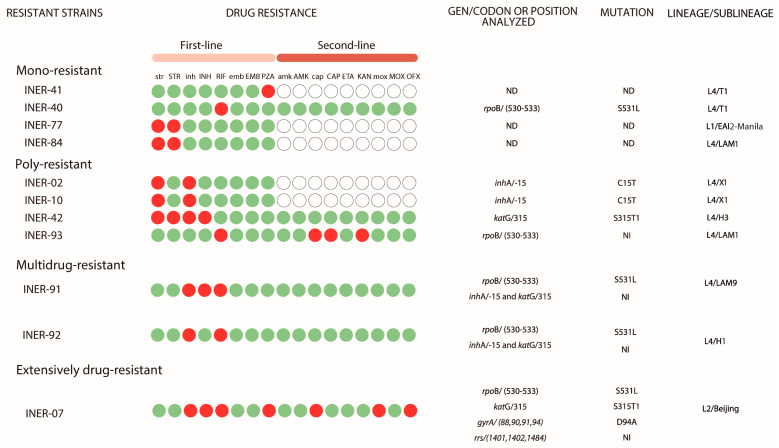
Resistance patterns, associated mutations, and lineage and sublineage to which the strain belongs. str: Streptomycin (1.0 μg/mL); STR: Streptomycin (4.0 μg/mL); inh: Isoniazid (0.1 μg/mL); INH: Isoniazid (0.4 μg/mL); RIF: Rifampicin (1.0 μg/mL); emb: Ethambutol (5.0 μg/mL); EMB: Ethambutol (8.0 μg/mL); PZA: Pyrazinamide (100 μg/mL), amk: Amikacin (1.0 μg/mL); AMK: Amikacin (2.0 μg/mL); cap: Capreomycin (2.5 μg/mL); CAP: Capreomycin (5.0 μg/mL); ETA: Ethionamide (5.0 μg/mL); KAN: Kanamycin (2.5 μg/mL); mox: Moxifloxacin (1.0 μg/mL); MOX: Moxifloxacin (2.0 μg/mL); OFX: Ofloxacin (2.0 μg/mL). Green circle: SENSITIVE; red circle: RESISTANT; blank circle: was not performed. ND: not determined. NI: not identified. L1: Lineage 1 (Indo-Oceanic); L2: Lineage 2 (East-Asian); L4: Lineage 4 (Euro-American).

Drug susceptibility testing was performed for all *M. tuberculosis* strains for the five first-line anti-TB drugs (STR, INH, RIF, EMB, and PZA). A total of 11/93 (11.8%) strains were resistant to one or more drugs tested. Although most of the strains came from Mexico City, only 3/56 (5.3%) were resistant, compared to 7/28 (25%) resistant strains from the State of Mexico (Figure 1). Of the ninety three strains tested, eighty two (88%) were pan-susceptible, four (4%) were mono-resistant, four (4%) were poly-resistant, two (2%) were multidrug-resistant (MDR), and one (1%) was extensively drug-resistant (Table 1 and Figure 1). Of the four mono-resistant strains, one was resistant to PZA, one to RIF, and two resistant to STR. Regarding the four poly-resistant strains, three were resistant to STR and INH, and one was resistant to RIF, CAP, and KAN. The two MDR strains were resistant to INH and RIF. Finally, the strain classified as extensively drug-resistant was resistant to INH, RIF, PZA, CAP, MOX, plus OFX (Figure 2).

### 3.3. Mutations Identified in the Drug-Resistant M. tuberculosis Strains

The frequencies of gene mutations in *Mycobacterium tuberculosis* resistant strains identified by the GenoType MTBDRplus and GenoType MTBDRsl tests are shown in Figure 2. Of the five RIF-resistant strains, in one strain, the mutation was not detected, whereas in the remaining four strains, mutation *rpoB* S531L was identified. Regarding the six INH-resistant strains, in two strains, the mutations were not detected. In two strains, mutation *inhA* C15T was identified, whereas mutation *katG* S315T1 was identified in the other two strains. Finally, in the extensively drug-resistant strain, mutation *gyrA* D94A was also identified.

### 3.4. Spoligotyping

For the determination of lineage and sublineage, all drug-resistant *M. tuberculosis* strains identified in this study were spoligotyped. The sublineages obtained were T, LAM, X, H (Lineage 4), EAI (Lineage 1) and Beijing (Lineage 2) (Figure 2).

## 4. Discussion

The early diagnosis of drug-resistant TB and HIV, the prompt initiation of appropriate second-line anti-TB drugs and antiretroviral treatment (ART), sound patient support, and strong infection control measures are all essential components in the management of drug-resistant TB in people living with HIV [9]. Multidrug-resistant TB originates from the selection of mutations in *M. tuberculosis* during first-line anti-TB treatment, leading to resistance to RIF and INH. If inadequately treated, the further selection of mutations conferring resistance to fluoroquinolones and second-line injectables results in extensively drug-resistant TB and eventually resistance to all effective drugs [16]. In this study, 93 *M. tuberculosis* strains from the same number of HIV-infected patients were tested for drug-resistance patterns to first- and second-line anti-TB drugs and to determine the mutations related to drug resistance.

Of the 93 patients, 68 (73%) had extrapulmonary TB. In immune-compromised patients, *M. tuberculosis* may disseminate to different parts of the human body because of immunity deterioration [17]. Extrapulmonary TB most commonly occurs at sites such as the lymph nodes, pleura, bones and joints, the central nervous system, ocular, pancreatic, and skin tissues, and the genitourinary tract [18], as occurred in this study.

Most patients included in this study were male. In a recent meta-analysis, Wondmeneh and Mekonnen [19] found that in an HIV-positive population from Sub-Saharan Africa, the male gender (AHR = 1.43, 95% CI: 1.22–1.64) was a risk factor for TB. The CD4 T-lymphocyte levels are the main markers for disease severity in patients with HIV and the best markers yet for disease progression [20]. Most HIV-infected patients (73%) had extrapulmonary TB and most (94%) patients with extrapulmonary TB had a CD4 T-lymphocyte count < 350 cells/mm^3^; in contrast, most (78%) of the patients with pulmonary TB had a CD4 T-lymphocyte count > 350 cells/mm^3^. Extrapulmonary TB constitutes approximately 15%–20% of all TB patients but accounts for 50% among HIV-coinfected patients [21]. The essential role of CD4 T-lymphocytes in the control of mycobacterial infection has been highlighted in knockout mice [22]. Mice deprived of CD4 T-lymphocytes at different stages of infection showed disorganization of the granulomatous lesions [23]. Deletion experiments in the in vitro model of human granuloma have suggested that CD4 T-cells constitute the only T-cell population critical for granuloma formation [22]. Some studies have demonstrated that among HIV-infected patients with TB, having extrapulmonary TB—compared to pulmonary TB—was associated with lower CD4 T-lymphocyte counts [24,25,26]. Furthermore, severe immunosuppression (low CD4 T-lymphocyte counts), a characteristic of advanced HIV infection, increases the odds of having extrapulmonary TB versus pulmonary TB alone [27].

TB–HIV coinfection has always been associated with high rates of TB drug resistance [28]. In this study, an 11.8% drug resistance in *M. tuberculosis* strains was identified. Our value is lower than that reported by Lopez-Alvarez and colleagues [29], who reported that 39.6% (19/48) of the *M. tuberculosis* strains isolated from HIV-infected patients in Mexico showed resistance to one or more first-line anti-TB drugs, with 2% multidrug-resistant strains. In México, Munro-Rojas et al. [30] and Ordaz-Vazquez et al. [31] reported that 25% and 19% of the *M. tuberculosis* strains from patients diagnosed with pulmonary TB were resistant to at least one first-line drug, respectively; as in our study, the main resistance was for INH. The low percentage of drug resistance in *M. tuberculosis* strains (11.8%) identified in this study, may be due to the fact that most of the strains were isolated from new TB cases (82.7%), and some authors have reported that the percentage of drug-resistant *M. tuberculosis* strains is lower in new cases than in previously treated cases [1,32,33]. Additionally, 73% of the strains were isolated from extrapulmonary samples and some studies have reported a low rate of drug resistance in extrapulmonary strains compared with strains recovered from respiratory specimens [34,35]. Interestingly, we identified one extensively drug-resistant strain. The presence of this type of strain has previously been reported in Mexico [30,36,37]. Extensively drug-resistant TB occurs as a consequence of the inadequate treatment of MDR-TB patients. Early identification of the resistance and diagnosis as well as careful treatment of the MDR-TB patient can help prevent XDR-TB [38].

As one of the most potent first-line anti-TB drugs, RIF serves as a surrogate marker for the detection of MDR-TB as >90% of RIF strains are also INH-resistant [39]. In the RIF-resistant strains identified in this study, mutation *rpoB* S531L was observed in 80% (4/5) of the strains, rendering this mutation the most frequent one. This is in agreement with previous reports that have established this mutation as the most frequent in RIF-resistant strains in Mexico and other countries [36,39,40,41,42].

A mutation in the *katG* gene and *inhA* promoter region is responsible for resistance to INH [43]. In this study, mutations conferring INH resistance occurred at a similar rate in the *inhA* promoter region and the *katG* gene. Moreover, the mutations found in *katG* had the same mutation point, S315T1, and the *inhA* promoter region was mutated at the C15T position. These mutations are frequent among INH-resistant strains. Reta and colleagues [44] published a meta-analysis which included 19 studies from Ethiopia. In this work, a total of 949 *M. tuberculosis* strains with INH resistance were identified, among which a higher proportion of mutations was detected in the *katG* gene (95.8%; 909/949) and the *inhA* promoter region (5.9%; 56/949). These mutations are also among the most frequently identified in INH-resistant strains in Mexico [42]. In our study, one strain was detected with mutation *gyrA* D94A, which has previously been reported in fluoroquinolones-resistant strains circulating in Veracruz, México [45].

The sublineages obtained of the drug-resistant *M. tuberculosis* strains were T, LAM, X, H (Lineage 4), EAI (Lineage 1), and Beijing (Lineage 2). These sublineages have been described in Mexico as part of the genetic diversity of *M. tuberculosis* [46]. The lineage that appeared most frequently in our results was Lineage 4, with different resistance phenotypes, this lineage has been associated with a high rate of MDR strains [47]. The Beijing sublineage has been described as having the highest rate of resistance [48] as occurred in our study.

A limitation of our study is that the GenoType MTBDRplus and GenoType MTBDRsl kits did not determine the mutations conferring resistance to STR and PZA. Additionally, mutations in the *rpoB* and *katG* genes and the *inhA* promoter region were not identified, making it necessary to use more robust methods, such as PCR, microarray, or whole genomic sequencing (WGS). This last tool can be used in the diagnosis of resistance against first- and second-line drugs; moreover, WGS allows the characterization of the genotypes in circulation and identifies transmission clusters, facilitating the further development of epidemiological-genomic surveillance studies [45].

In conclusion, our study shows that a percentage of *M. tuberculosis* strains isolated from HIV-seropositive patients was resistant to one or more drugs. Of note, one strain evaluated was extensively drug-resistant. Mutations associated with resistance were identified; however, in some strains, such mutations could not be identified, showing the need to use tools such as WGS. We highlight the need to perform drug susceptibility testing to provide adequate therapeutic management or expedite modification of TB treatment to decrease the risk of the spread and the transmission of MDR-TB and to reduce the number of deaths in HIV-infected patients.

## Figures and Tables

**Table 1:** Sociodemographic and clinical characteristics of HIV-infected patients and drug susceptibility testing of *M. tuberculosis* strains.

Variable		Total Cases (%)
Sex	Male	80 (86)
	Female	13 (14)
State of origin	Mexico City	56 (60)
	State of Mexico	28 (30)
	Veracruz	2 (2)
	Hidalgo	2 (2)
	Guerrero	2 (2)
	Puebla	2 (2)
	Morelos	1 (1)
Age (years)	≤36	49 (53)
	≥37	44 (47)
Occupation	Unemployed	31 (33)
	Employed	26 (28)
	Disabled	12 (13)
	Trader	12 (13)
	Another	12 (13)
Formal education	None or basic	64 (69)
	Intermediate or advanced	29 (31)
TB type	Pulmonary	25 (27)
	Extrapulmonary	68 (73)
Clinical sample	bronchoalveolar lavage fluid	17 (18)
	Sputum	15 (16)
	Lung biopsy	6 (6)
	Blood	15 (16)
	Bone marrow	11 (12)
	Urine	8 (9)
	Cervical lymph node	8 (9)
	Pleural effusion	6 (6)
	Subcutaneous abscess	4 (4)
	Cerebrospinal fluid	3 (3)
CD4 T-lymphocyte count < 350/mm^3^	Pulmonary ^a^	3 (6) ^b^
	Extrapulmonary ^a^	48 (94) ^b^
CD4 T-lymphocyte count > 350/mm^3^	Pulmonary ^a^	14 (78) ^c^
	Extrapulmonary ^a^	4 (22) ^c^
Resistance pattern	Pan-susceptible	82 (88)
	Mono-resistant	4 (4)
	Poly-resistant	4 (4)
	Multidrug-resistant	2 (2)
	Extensively drug-resistant	1 (1)

^a^ For whom data were available—not all data were available for all patients. ^b,c^ *p* ≤ 0.0011 using Fisher’s exact test.

## Data Availability

All data derived from this study are provided in the article.
